# Analysis of Filtration Coefficient of Selected Recycled Materials on the Example of Concrete Aggregate and Rubber Waste

**DOI:** 10.3390/ma18184240

**Published:** 2025-09-10

**Authors:** Katarzyna Gabryś, Karolina Damska, Raimondas Šadzevičius, Dainius Ramukevičius, Wojciech Sas, Bruno Camargo, Algirdas Radzevičius, Midona Dapkienė

**Affiliations:** 1 Department of Geotechnics, Institute of Civil Engineering, Warsaw University of Life Sciences—SGGW, 159 Nowoursynowska Street, 02-787 Warsaw, Poland; karolina.damska@gmail.com (K.D.); wojciech_sas@sggw.edu.pl (W.S.); 2 Department of Water Engineering, Vytautas Magnus University Agriculture Academy, 53361 Kaunas, Lithuania; dainius.ramukevicius@vdu.lt (D.R.); algirdas.radzevicius@vdu.lt (A.R.); midona.dapkiene@vdu.lt (M.D.); 3 Faculty of Physics, Institute of Experimental Physics, University of Warsaw, 5 Ludwika Pasteura Street, 02-093 Warsaw, Poland; bruno.camargo@fuw.edu.pl

**Keywords:** recycled concrete aggregate, recycled tire waste, permeability, filtration coefficient, variable gradient method

## Abstract

The permeability of recycled materials such as recycled concrete aggregate (RCA) and rubber tire waste (RTW) significantly affects their suitability in geotechnical applications. RCA is typically more porous than natural aggregates, while RTW can either increase or decrease permeability depending on its content and form. This study investigates the hydraulic conductivity of fine RCA (fRCA), fRCA–RTW mixtures, and compressed shredded tire waste (RTWS) using variable-gradient tests under various consolidation pressures. Permeability is closely related to material quality, depending on intended use: low permeability suits barrier or fill layers, while high permeability benefits drainage applications. Both behaviors were achieved in this study—fRCA showed low permeability (10^−6^ to 10^−7^ m/s), while RTW addition significantly increased water flow, with filtration coefficients exceeding 1 × 10^−3^ m/s. The permeability of fRCA–RTW mixtures increased with rubber content, though greater heterogeneity was observed. The results demonstrate that recycled materials can be tailored for specific hydraulic functions, supporting their use in sustainable construction.

## 1. Introduction

Over the past few years, an increase in the average global air temperature has been observed. This rise is evidence of climate change, which has already started to negatively impact the environment, human life, and the management of natural resources [1]. Along with human population growth, the technological development of non-renewable natural resources is gradually being depleted [2]. To keep our environment and planet in a better state, more attention is being paid to the use of natural soils and adherence to sustainable development goals [3]. Managing soil resources during construction processes, such as civil or hydraulic engineering, involves using various aggregates, including all-in aggregate, coarse aggregate, and discrete aggregate. These aggregates are classified as natural, non-renewable resources [4]. The simplest way to substitute is to find a material or mixture of materials that matches the properties of the material being substituted. The most popular method of tackling excessive pollution is recycling.

Recycling is considered to be the most effective method of preserving our planet’s environment. By simply reusing the material instead of throwing it away, we can decrease the amount of waste materials and also conserve future natural resources. This will reduce the amount of waste sent to landfills and help prevent water and air pollution [5]. Recycling in the construction industry focuses on reusing waste from construction and demolition. Construction and demolition waste are the waste materials that are produced in the process of construction, renovation, or demolition of residential or nonresidential structures. Components of construction and demolition waste typically include concrete, asphalt, wood, metals, gypsum wallboard, roofing, paper, plastic, drywall, and glass. Concrete is the second most consumed material after water; therefore, recycling concrete can save construction costs. Also, it will help to keep the environment healthy [6]. Concrete collected from sites is put through a crushing machine, usually uncontaminated concrete, i.e., free from wood, plastic, paper, and other unwanted materials, and crushed into aggregate with varying degrees and used in new projects. Depending on the requirements, it might be used alone or mixed with other materials or additives in various proportions.

Recycling in geotechnical engineering applications also applies to the recycling of discarded tires [7]. Reusing scrap tires has become necessary to reduce their impact on the environment and public health. Recycling scrap rubber helps to save large amounts of energy, which ultimately reduces greenhouse gas emissions. Recycling four tires is estimated to reduce CO_2_ emissions by around 146 kg, the equivalent of 69 L of petrol. Beyond the environmental benefits, recycling waste rubber plays an important role in stimulating the economy and creating jobs. It is, therefore, reasonable to investigate scrap tires as a potential geomaterial for various construction applications [8].

The properties of substitutes, or in other words, alternative materials, should be tested before application in any structural elements. Skipping this particular step might lead to a construction disaster during the building phase and later during constant use [9]. In the construction industry, alternative aggregates are mainly used in earth and road construction [10]. The material to be incorporated into earth/road embankments must meet technical, economic, and environmental requirements. Key parameters include grain size, strength, compaction, durability, and especially permeability, which is critical for ensuring stability in structures exposed to water [11]. However, assessing these parameters is often challenging due to the lack of standardized testing methods for recycled or waste-derived materials, variability in material quality, and limited long-term performance data [12]. Differences in source composition, inconsistent processing, and non-traditional behaviors—such as non-Darcian flow in rubber-modified mixtures—further complicate evaluation. These limitations hinder widespread acceptance and may pose risks if not properly addressed through thorough testing and regulation. Proper analysis ensures safety in projects, especially dams, tunnels, or building foundations exposed to water’s harmful and sometimes destructive effects [13].

Soils are permeable materials due to the presence of interconnected voids that allow fluids to flow from high-energy to low-energy locations. The correct evaluation of soil permeability is necessary for the calculation of seepage under hydraulic structures and water volumes during dewatering activities. Soil permeability is influenced by several factors, including void ratio, distribution of intergranular pores, and degree of saturation [14]. The presence of groundwater on a building site often complicates the execution of work and requires additional intervention using special equipment. The ability of the soil to conduct water is a key factor in the consolidation process, as it determines the intensity of this phenomenon. In almost all cases, the construction of buildings is directly or indirectly linked to water flow in the ground. Therefore, the problem of water flow in soil has been the subject of scientific research for many years [15].

Several methods are used to determine the flow parameters of soils. The methods for determining percolation parameters described in the literature can be divided into two groups: computational (theoretical or semi-empirical) and research (experimental) methods. Examples of computational methods are analytical and empirical formulae, numerical modeling, and mathematical inverse solutions. Within experimental methods, we can distinguish between laboratory tests on undisturbed (NNS) or disturbed (NS) samples and in situ tests (field tests). These methods can be categorized into indirect and direct tests [16].

The investigation examines the hydraulic conductivity using the permeameter method, according to ASTM procedure D5084 [17], and employing a self-developed permeability test device by the Lithuanian team, in the case of pieces of shredded tires. Table 1 summarizes selected previous studies on the water flow characteristics of tested waste materials. The literature search reveals little information on the permeability of pure rubber waste and no mention of its filtration coefficient when mixed with RCA. Thus, the novel aspects of this article are as follows: (i) the determination of the *k* parameter of compressed pure tire shreds and (ii) the inclusion of waste mixtures in the permeability research.

The objective of this article is to characterize the coefficient of permeability (k) of selected recycled materials. The permeability coefficient varies over a wide range of up to 10 orders of magnitude from coarse to very fine-grained soils [34]. The present study investigates the filtration of recycled tire waste shreds (RTWS), fine recycled concrete aggregate (fRCA), and mixtures composed of two waste materials: fRCA and recycled tire waste powder (RTWP) and recycled tire waste granulate (RTWG). This approach is an ecological and sustainable alternative to waste disposal, particularly for rubber waste, which is difficult to decompose and can pose an environmental problem.

## 2. Materials and Methods

The materials used in this work are fine recycled concrete aggregate, which is mixed with rubber granulate from waste car tires and shredded tires. Their characteristics are summarized below.

### 2.1. fRCA

The main raw material employed in the present study and in two fRCA–RTW mixtures was fine recycled concrete aggregate from the crushed concrete curbs from the demolition of roads in Warsaw, the capital of Poland. As defined, fRCA (particles < 4.0 mm) are derived from multiple crushing of concrete debris [35]. Fine RCA, which contains most of the hardened paste, is typically recycled and used in low-value applications as opposed to coarse fractions (particle size > 2–4 mm) used for high-end valorization [36].

**Figure 1:**
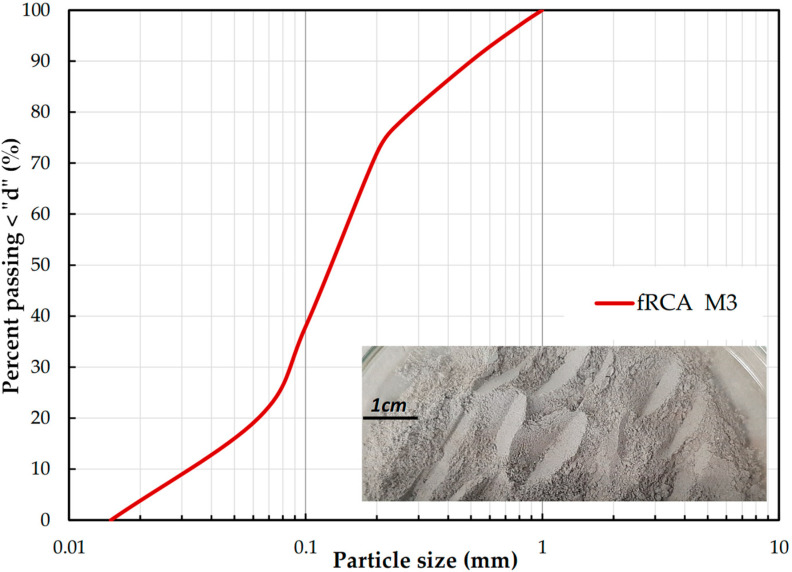
Particle size distribution of the M3 mixture and its photograph.

The production of this test material in the geotechnical laboratory consisted of additional crushing of the material to obtain specific grain fractions < 0.063 mm, 0.063–0.125 mm, 0.125–0.25 mm, 0.25–0.50 mm, 0.50–1.0 mm, 1.0–2.0 mm [35]. From these fractions, mixed in appropriate proportions, several specific compositions were obtained. One of them, termed in the previous authors’ research as M3 [37], was selected for the current permeability tests. The sum of crushed concrete, mortar, and unbound natural aggregate in it was more than 90%. The remainder, less than 1%, consisted of glass and brick. No asphalt or tar elements were noted. The soil gradation curve (Figure 1) was designed using fractions ranging from 0.015 to 0.8 mm, based on mass percentage. This composition was achieved by carefully mixing two materials: pure fRCA and the calculated amount of fine fraction (FF) content of fRCA in the quantity of 20%. The mixing process involved shaking the materials in a closed container. A photograph of the M3 mixture is given in Figure 1. The resulting mixture was categorized as uniform SAND with silt, based on grain size ratios, such as the coefficient of uniformity (C_U_) and curvature (C_C_), see [38] for details. The strength class of the concrete, along with its selected physical and chemical properties, was estimated prior to the permeability tests. The results of these tests are presented inter alia in [37,38,39].

### 2.2. fRCA, RTWP and RTWG

Shredded tire material, free of steel, wires, and fibers, was collected from a Polish local tire recycling company—Orzeł SA. Orzeł SA is engaged in the ecological production of rubber granules in Poniatowa, Poland. They offer products made from recycled tires. Their granules are a modern alternative to traditional surface materials. The company provides products with high purity that meet safety standards.

**Figure 2:**
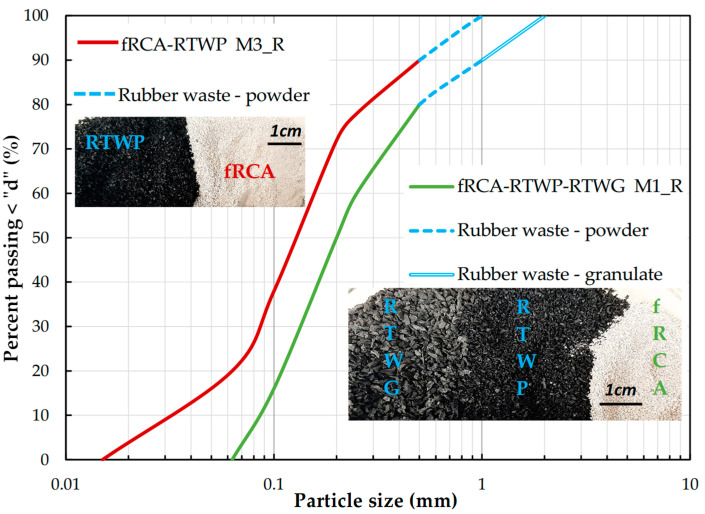
Particle size distribution of the M1_R and M3_R mixtures and their photograph; right—M1_R with powder and granulate rubber waste; left—M3_R with powder rubber waste.

In our research, two sizes of tire waste were used: powder (P) with a diameter size of 0.5–1.0 mm (photograph in Figure 2, left) and granulate (G) with dimensions of 1.0–2.0 mm (photograph in Figure 2, right). The desired tire particle size was obtained by appropriate sieving in the geotechnical laboratory. Powder granules were produced exclusively from light vehicle tires. The second type of granules, which are thicker, are named BASE granules, made from 80% truck tires. A detailed description, including a technical card, granulometric analysis, and the most common applications for each fraction, can be found on the manufacturer’s website. Some characteristics are also available in the other authors’ article [39].

To investigate the permeability parameters, two different compositions were prepared, varying the target rubber waste content (%) to 10% and 20% by dry mass. The mixtures were named according to the fRCA matrix: M1_R and M3_R. They have the following compositions:

- M1_R–fRCA with 10% addition of RTWP, 10% addition of RTWG, and 0% of FF content of fRCA,
- M3_R–fRCA with 10% addition of RTWP and 20% of FF content of fRCA.

Particle size distribution curves of the tested blends are shown in Figure 2 with photographs. The blend M3_R, in terms of grain size, corresponds to a mixture with rubber powder, i.e., fRCA_M3. The M3_R mixture was created by replacing the 0.5–1.0 mm crushed concrete aggregate in the fRCA_M3 mix with rubber waste of the same size. In the case of the M1_R mixture, it was classified as poorly graded fine SAND (C_U_ = 3.02, C_C_ = 0.88), following the classification of natural soils [40].

### 2.3. RTWS

**Figure 3:**
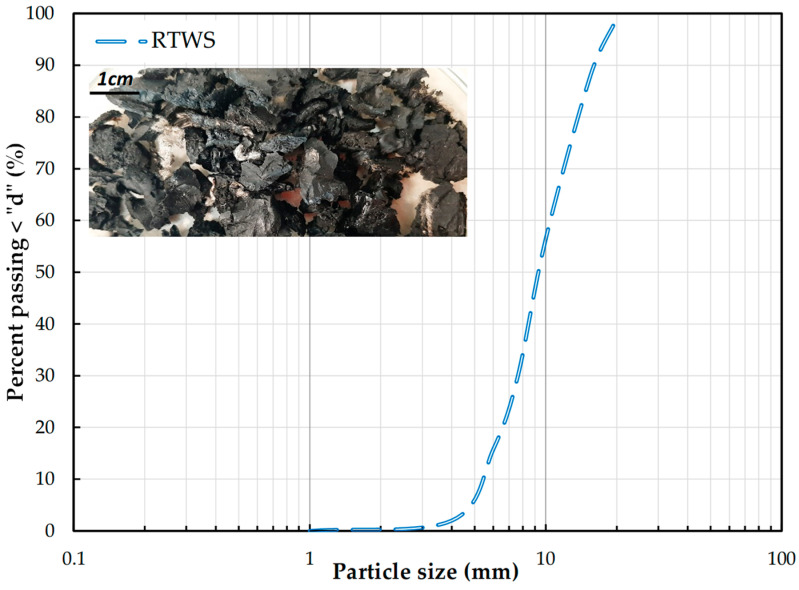
Particle size distribution of the recycled tire waste shreds and their photograph.

A Lithuanian company called JSC “Dormeka”, Didžiasalis village., Vilnius district, Lithuania has suggested using its waste tires to test how effective they would be as landfill drainage layers. The company is affiliated with the Engineering Ecology Association. These are pieces of tires, called tire shreds, composed of approximately 80% truck tires. Due to their size, they can be used as a base layer for sports surfaces, in the production of rubber tiles and mats, and are rarely used as a supplement to artificial grass. They may also be used in gardening, landscaping, and animal husbandry. Tires of coarser processing were cut into smaller pieces. These measured up to 15 cm. However, in the presented studies, only pieces measuring a maximum of 20 mm were used (Figure 3). The material obtained from Dormeka was carefully sorted and 99% cleaned of metal and other unwanted impurities. Alternatively, approximately 1% may contain textile cord. RTWS gradation ranges from ~1.0 mm to ~20 mm, and the grain size ratios, C_U_ = 1.9, C_C_ =1.03, indicate homogeneous, poorly graded material, which corresponds to gravel in terms of grain size.

### 2.4. Equipment and Testing

**Figure 4:**
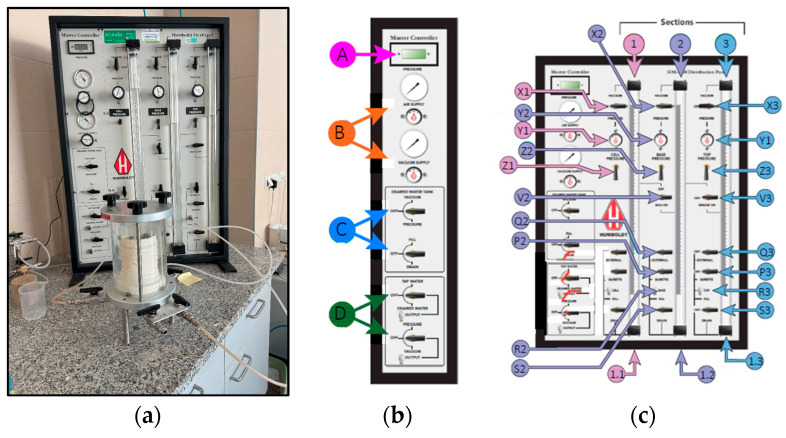
(**a**) Testing setup in SGGW, Warsaw, Poland; (**b**) the master controller; (**c**) HM-4150 with one group of control burettes labelled for reference (based on HUMBOLDT Mfg. Co., Elgin, IL, USA [34]).

In this paper, for permeability testing, two different pieces of equipment were used. The permeability coefficient of the three tested mixtures: M3, M1_R, and M3_R, was investigated using the Humboldt Flex Panels model “HM-4150” (Elgin, IL U.S.A), called in this study as permeameter) (Figure 4a). This model is part of the equipment of the geotechnical laboratories of the Warsaw University of Life Sciences (SGGW), Warsaw, Poland. The panel is utilized for controlling the pressures and monitoring the flow of water in and out at various effective stresses and hydraulic gradients. In Figure 4b, the different sections of the master controller are highlighted as A, B, C, and D. Part A allows the display of the current pressures (cell, base, or top) controlled by toggle valves. Part B regulates air and vacuum pressures supplied to the Flex Panel, using adjustable pressure regulators and gauges. Parts C and D, on the other hand, control the de-aired water tank, manage vacuum, water flow, and connections to the waste drain or water supply. In addition, they are responsible for the administration of water supplies, compressed air, and vacuum systems for the apparatus [41]. Humboldt Flex Panel controllers are divided into groups, each containing three control burettes that can independently regulate pressure or volume changes. The system is shown in Figure 4c. Section 1 controls the main chamber of the triaxial cell and manages the external water pressure. It regulates the applied effective stress, facilitating the consolidation process. Section 2, also referred to as the Base section, controls the flow of water into and out of the bottom of the sample to ensure proper control during testing. Section 3 (the Top section) regulates the flow of water into and out of the top of the sample, maintaining precise control of the upper part during experiments. Section valves (X1, X2, and X3) determine the type of pressure input for their respective sections (vacuum, atmospheric, or regulated pressure). Pressure regulators (Y1, Y2, and Y3) provide controlled pressure to the burette assemblies in their respective sections. Toggle valves (Z1, Z2, and Z3) are used to monitor pressure readings in their respective sections. They connect directly to the pressure regulators to enable precise adjustments. External valves (Q2 and Q3) connect to the external ports of their respective burette assemblies, facilitating water flow and pressure adjustments. External quick-connect ports (R2 and R3) link the burette assemblies to external systems. S2 and S3 are fill/drain valves used to fill or drain the burette assemblies in Section 2 and Section 3. They control the flow of water or other fluids into or out of the burette system.

**Figure 5:**
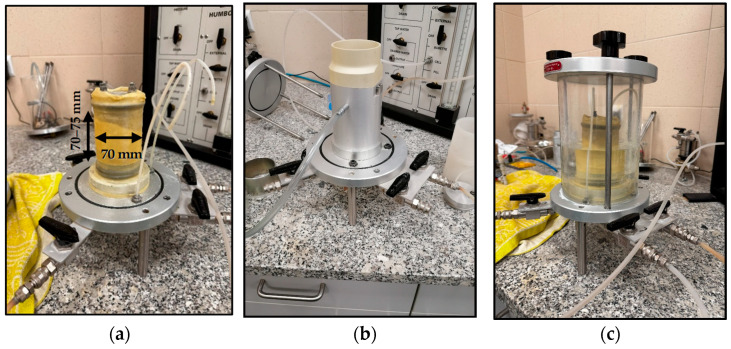
(**a**) Cylindrical sample prepared from crushed concrete; (**b**) cylindrical mold with soil sample; (**c**) soil sample on the permeameter base.

The entire measurement process in HM-4150 was based on creating a pressure difference between the top and bottom of the soil sample to cause water to flow. Burette Section 2 and Section 3 were used primarily. Before preparing the sample, it was necessary to ensure all tubes and connections/transitions are sealed and unobstructed, so the water flows freely to the sample and drains from it. For this study, a series of specimens measuring approximately 70 mm in diameter and ~70–75 mm in height (as specified by the manufacturer) (Figure 5a) were prepared using the dry tamping method. The constant-volume split cylindrical mold (Figure 5b) was used to cast the cylindrical sample. To achieve a uniform density, the under-compaction effect was induced by the energy transmitted when subsequent layers were tamped during specimen preparation [42]. Specimens were tamped in five equal layers (thickness ≈ 1.4 cm) at a free drop height of 10–50 mm. The number of blows varied to produce homogeneous samples. Particular attention was paid to avoiding any particle segregation. The previously prepared mixture was then placed in a split mold and carefully compacted at a predetermined height to achieve the target density. Compaction was performed manually using a graduated vertical rod and a small removable horizontal tab that controlled the desired height by abutting against the edges of the mold. The target compaction level was set at 90%. In Figure 5c, the typical granular soil sample enclosed in the permeameter cell is presented. A step-by-step procedure for preparing the non-cohesive sample in this type of apparatus is included, for example, in the work of Kandalai et al. [43].

The testing procedure adopted in this study included stages like flushing, allowing water to flow through the ‘top’ and ‘base’ tubes to remove air from the lines, as well as any air trapped between the membrane and the soil. The process was considered complete when no more bubbles were visible in the ‘top’ and ‘base’ tubes, or the tubes connecting the ‘cell’ with the flex panels. All the mixtures were subsequently saturated with de-aired water by maintaining a pressure difference between the inside of the permeameter chamber (~210 kPa) and the soil sample (~200 kPa). A pressure difference of 10 kPa was sufficient for this process and small enough to avoid changes in the sample’s density. The saturation stage took about 24 h. The final stage in preparing for proper permeability testing was consolidation at various effective stresses (*p’* = 10–120 kPa). Pressure was increased until the target effective stress was reached, after which the final test—the constant head hydraulic conductivity test—could begin. It was very important to start with the lowest effective stress and then increase the pressure as needed. To obtain different effective stresses, a specific pressure on the ‘top’ of 200 kPa was applied, and different variable, increasing pressures were applied in the ‘cell’ (referred to as confining pressure, σ_3_). Each consolidation stage lasted 24 h. The experimental plan is listed in Table 2.

In the present permeability studies using a permeameter HM-4150, the variable gradient method was used. Each of the following mixtures (i.e., M3, M1_R, and M3_R) was tested at different, increasing hydraulic gradients (*i* ≅ 1–16) (Table 2), by creating a difference in pressures, so called input flow pressure (f_p_) applied at ‘top’ (always equal to 200 kPa) and ‘base’ (always over 200 kPa) of the sample (Table 3). Each test consisted of measuring the amount of water (Q) that flowed through the sample in a specified time. The flow of water was recorded at intervals of 5–10 min until a steady-state flow condition was achieved. It was observed that the time required to reach a steady-state flow was approximately 1–1.5 h. Each *Q* measurement was repeated 4–5 times while maintaining the same gradient and effective stress. The idea was to minimize the chance of error and ensure accurate results. Therefore, in the HM-4150 test, one permeability experiment, at a given *i* and *p’* lasted at least 2 h.

This structured approach ensures consistent testing at all stress levels, providing a comprehensive dataset for analyzing how the material behaves under different gradients and pressures.

The hydraulic conductivity (*k*) of granular material, fRCA and RCA–RTW mixtures, under different *p’* and varying *i* was computed using Equation (1):

(1) $$k=(\frac{Ql}{∆hA}),$$

where *Q* = discharge flow rate ($\frac{m^{3}}{s}$), *i* = hydraulic gradient $\left(\frac{∆h}{l}\right)$, *l* = sample height (m), Δ*h* = pressure head $\left(\frac{f_{p}}{\gamma_{w}}\right)$, *f_p_* = input flow pressure (kPa), *γ_w_* = unit weight of water $\left(\frac{kN}{m^{3}}\right)$, and *A* = area of sample (m^2^). The *k* parameter calculated from Equation (1) is applied to the given temperature of the flowing water. In the presented studies, it was *T* = 23 °C. Hence, the next step was to calculate the hydraulic conductivity at a water temperature of 10 °C (*k*_10_) following Equation (2):

(2) $$k_{10}=\frac{k(T)}{\left(0.7\;+\;0.03T\right)},$$

where *k*(*T*) = filtration coefficient obtained for water at a temperature *T* ($\frac{m}{s})$ and *T* = temperature of flowing water during tests (°C).

**Figure 6:**
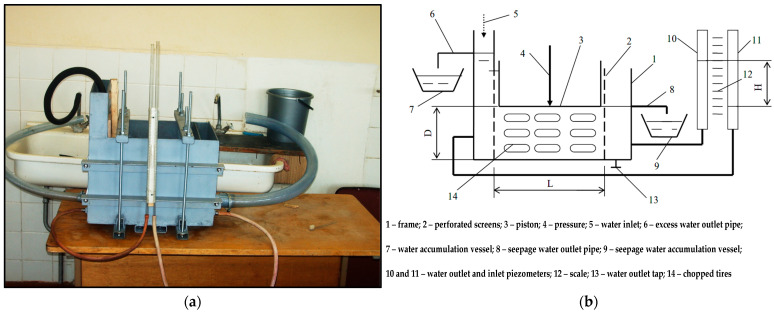
(**a**) Testing setup in VMU, Kaunas, Lithuania; (**b**) configuration of the instrument for the water permeability assessment.

The permeability coefficient of the shredded tires was investigated at the Vytautas Magnus University Agriculture Academy (VMU), Kaunas, Lithuania, employing the homemade apparatus, presented in Figure 6a. The principle diagram of this device is displayed in Figure 6b.

Its main parts are made of sheet steel, and the joints are welded. Steel surfaces are covered with an anti-corrosive paint coating. The parameters of the device’s RTWS-filled section are selected based on the thickness of the waste material. In the presented study, the layer of compressed shredded tires was 5–10 times thicker than the average piece. The diameter of the piezometers was chosen so that they would show the water levels at the inlet and outlet without delay.

**Figure 7:**
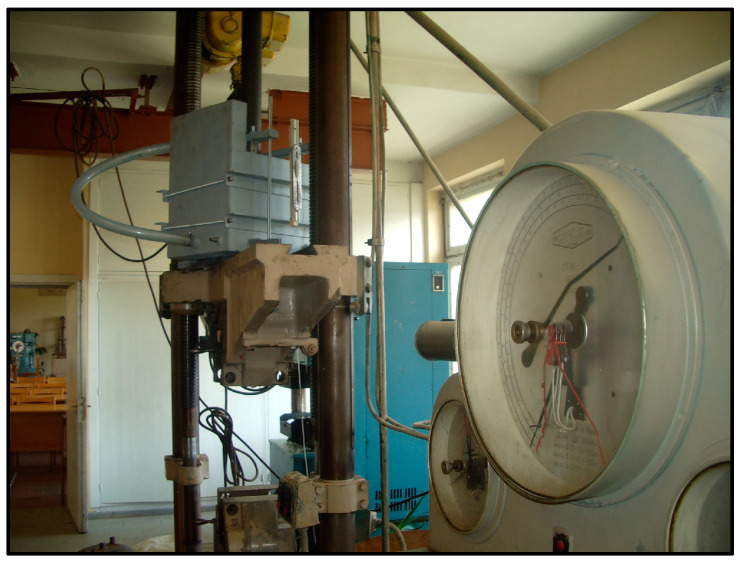
Compression of chopped tires by a hydraulic testing machine (Vytautas Magnus University Agriculture Academy (VMU), Kaunas, Lithuania).

This device operated according to the following principle. The shredded rubber (14) (hereinafter referred to as RTWS) was poured into the filtration device container, which was enclosed by two water-impermeable walls and a bottom, and two perforated walls (2), after the piston (3) had been lifted. RTWS were placed in 10 cm layers, which were levelled and compacted using a wooden rod with a 4 cm square cross-section. A layer 45 cm thick was formed. Subsequently, water was added to the device until it was above the top of RTWS. The rubber was then compressed to the required pressure using a hydraulic press (Figure 7). The pressure was kept consistent with a precision of 5%. The shredded rubber was compressed above the piston (3), and the water drained via the valve (13). The piston plate and body of the device were sealed together. After opening the water tap, water was supplied through the hose to part (5) of the device. The excess water was expelled through the drain pipe (6). The flow of water through the drain pipe (8) began when the layer of crushed rubber was filtered. Once the flow had been stabilized, the pressure height should have been determined using the pressure gauges and scale (12) for the water supply (10) and drain (11).

In the case of RTWS, the measurements concerned the longitudinal filtration. Before starting the tests, the rubber was compressed sufficiently. This ensured that water flows freely through the filtrate outlet pipe (8). The level of the rubber was not higher than the connection point of this pipe to the device body (1). It was also imperative to verify that the water level in the water supply section (5) did not exceed the connection point of the excess water outlet pipe (6) to the device body (1). The water pressure level was checked again using the piezometers (10, 11) and the scale (12). The thickness (D), width (B), and length (L) of the compressed rubber layer, as well as the water temperature, were measured with millimeter accuracy. Next, the filtrate collection container (9) was emptied, and the time measurement began with a stopwatch. The water filtration was determined in 3–4 time intervals, each lasting 5–10 min.

A total of three samples of shredded tire pieces from JSC “DormekaDidžiasalis village., Vilnius district, Lithuania), referred to here as RTWS1, RTWS2, and RTWS3, with dimensions listed in Table 4, were examined. During the filtration tests, the hydraulic gradient from the lowest possible *i* ≅ 0.05 to the highest possible *i* ≅ 0.3–0.8 was changed. The chosen hydraulic gradient is typical for water damming construction [44]. For each gradient *i,* from 3 to 5 measurements of the parameter *k* were performed. The compression pressures (*p’*) varied in the range from 0 to around 1000 kPa. For the pieces of tires poured into the cylinder, the required pressure was transmitted through a rigid steel disc (Figure 7).

The hydraulic conductivity (*k*) of shredded tires, RTWS, was computed using Equation (3):

(3) $$k=(\frac{Vl}{HArt}),$$

where *V* = volume of filtered water determined by volume using metrologically verified measuring vessels, or by weight ($m^{3}$), *l* = filtration path (distance between perforated partitions) (m), *H* = water pressure head (difference in water levels in piezometers) (m), *A* = area of sample (m^2^), *r* = temperature correction coefficient, which is used to estimate the temperature of the flowing water when it is lower or higher than +10 °C, *t* = filtration time (s). The temperature correction coefficient (*r*) was calculated based on Equation (2), as follows:

(4) r=0.7+0.03T,

where *T* = temperature of flowing water during tests (°C).

## 3. Results and Discussion

### 3.1. fRCA–RTW Mixtures

**Figure 8:**
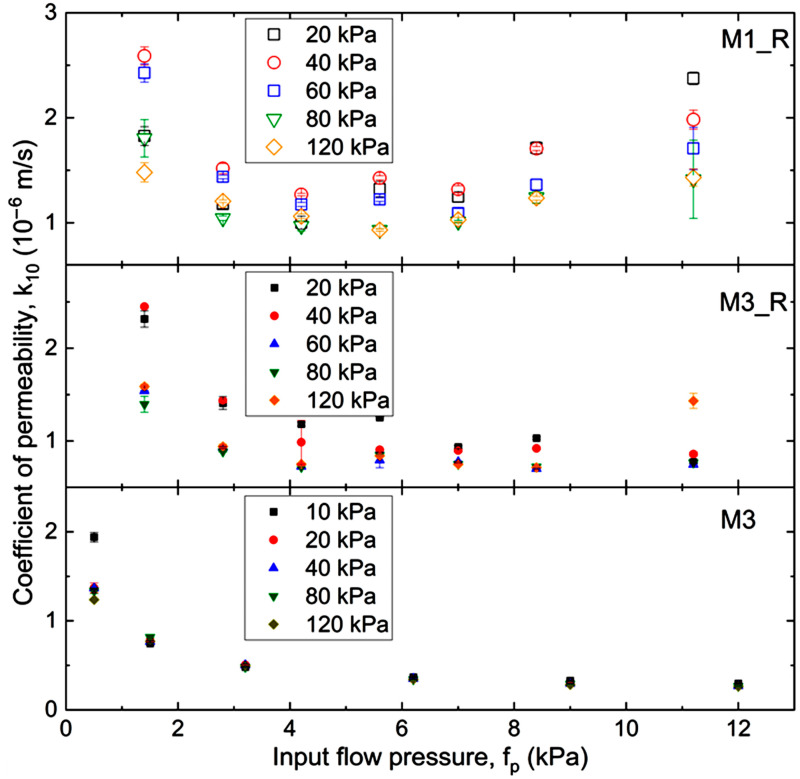
Variation in *k*_10_ with *f_p_* corresponding to varied *p*’ for the tested mixtures (*p*’-values are given in the legend; *k*_10_ data are shown with the error bars); top panel—M1_R mixture; middle panel—M3_R mixture, base panel—M3 mixture.

In Figure 8, the variation of the permeability coefficient is plotted with the input flow pressure. The filtration coefficient values presented were calculated for a water temperature of 10 °C. For each measurement, the standard error was also calculated, marked on Figure 8 with error bars, symbolizing the uncertainty surrounding each estimate.

For the mixtures containing rubber waste (M1_R and M3_R), the values of *k*_10_ range from approximately 0.8 × 10^−6^ to 3.0 × 10^−6^ m/s. For the M3 mixture, which contains only fine recycled concrete aggregate (RCA) without rubber, lower permeability was obtained, with *k*_10_ values ranging from approximately 0.3 × 10^−6^ to 1.8 × 10^−6^ m/s. The fine fraction of RCA enhances compaction and inter-particle adhesion, reducing porosity and, therefore, permeability (Figure 8, bottom panel, M3). Similar values have been reported in the literature: Azram and Cameron [45] reported *k* coefficients of 2.0 × 10^−7^ to 2.0 × 10^−8^ m/s for RCAs (0–20 mm, 6–7% fines), Arulrajah et al. [46] obtained 2.04 × 10^−3^ to 3.30 × 10^−8^ m/s using constant head tests, and McCulloch et al. [47] found 1.0 × 10^−4^ to 3.0 × 10^−4^ m/s for RCAs with 4% fines and particle sizes of 0–50 mm.

The incorporation of rubber waste in M1_R and M3_R increases permeability relative to M3. This occurs because rubber particles create additional voids and interconnected flow paths within the RCA matrix. The influence depends on both the quantity and the type of rubber used. The incorporation of 5% of RTW does not cause an increase in overall porosity [48]. A closer look at Figure 8 (middle panel) reveals that, for example, 10% RTWP in M3_R increases the permeability coefficient by an average of ~53% compared to M3. Similarly, adding 10% of coarser rubber particles (RTWG) in M1_R leads to a further increase of ~25% compared to M3_R (Figure 8, top panel). The increase arises not only from the greater proportion of rubber, but also from the larger particle dimensions [49].

Based on the results, the studied fRCA–RTW mixtures are characterized by *k_10_* values corresponding to poorly permeable silty and clayey sands (M1_R and M3_R), while M3 behaves more like semi-permeable clays [50].

For the M3 mixture (bottom panel), permeability decreases significantly as *f_p_* increases up to ~6 kPa, after which *k*_10_ stabilizes and remains nearly constant. This suggests that at low pressures, finer RCA particles migrate into voids between coarser particles, reducing porosity and permeability [51,52,53]. This trend is well described by a power function up to *f_p_* ≈ 6 kPa (R^2^ = 0.98):

(5) $$k_{10}=0.9603f_{p}^{-0.531}.$$

Beyond this threshold, permeability is effectively pressure-independent. This behavior is consistent with findings of other researchers, who observed permeability reductions with consolidation pressure, although in this case the applied flow pressures are relatively low (≤120 kPa), which may explain the limited changes in *k* [54].

For the rubber-modified mixtures, a similar tendency of decreasing *k*_10_ with increasing *f_p_* is observed, especially in M3_R with 10% rubber. A deviation occurs at *f_p_* = 20 kPa, likely due to experimental errors such as incomplete saturation or air bubbles in the flow lines. At higher pressures (≥60 kPa), the relationship between *k*_10_ and *f_p_* becomes weak, and permeability values tend to stabilize.

The M1_R mixture (top panel) shows greater variability than M3 or M3_R. At lower pressures (*f_p_* ≤ 5 kPa), permeability decreases due to pore rearrangement and partial closure of voids. At higher pressures, however, an upward trend in permeability appears. This may be attributed to the activation of macropores associated with the rubber particles, leading to increased flow. The data suggest two behavioral regimes: aggregate-dominated flow at low *f_p_*, and rubber-dominated flow at high *f_p_*. For M3_R, this transition occurs at slightly higher pressures (~8 kPa), while for M3 (no rubber), it is absent altogether.

Finally, as the mean applied pressure increases, the permeability of M1_R generally decreases due to consolidation of the rubber particles. Compression of the tire fragments reduces the void ratio and the connectivity of pore spaces, diminishing the material’s ability to transmit water. This highlights the complex role of rubber waste in RCA mixtures: it can both enhance permeability through macropore formation and reduce it through compression effects.

**Figure 9:**
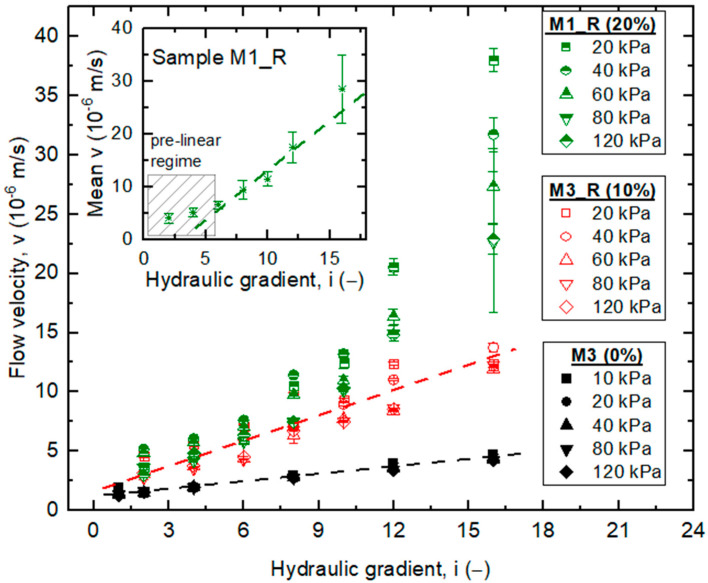
Relationship between *v* and *i* for the tested blends (*p*’-values are given in the legend; *v* data are shown with the error bars; green color indicates data for 20% rubber content, red—10%, black—0%).

The velocity of water flow through the tested recycled materials, which was calculated using Darcy’s Law, according to Equation (6):

(6) $$v=\frac{Q}{A},$$

is plotted with the hydraulic gradient in Figure 9. As expected for porous media, flow velocity increases with hydraulic gradient for all tested mixtures, whether pure recycled concrete aggregate (M3) or modified with rubber waste (M3_R, M1_R).

The rate of increase is notably higher for mixtures containing rubber. For example, at *i* ≈ 16 and under the lowest confining pressure, the average flow velocities are approximately 5.2 × 10^−5^, 1.9 × 10^−5^, and 0.6 × 10^−5^ m/s for M1_R, M3_R, and M3, respectively. Thus, the greater the amount of rubber incorporated, the higher the resulting flow velocity.

At higher gradients (*i* ≥ 8), the differences between the mixtures become more pronounced. For example, the velocity difference (Δ*v*) between M3 and M1_R ranges from ~20% at lower gradients to over 70% at the highest tested gradients. Consolidation pressure also influences the results: increasing pressure reduces flow velocity by approximately 15–30%, depending on the magnitude of the pressure applied. This effect is more evident in the rubber-modified mixtures, where the deformability of rubber particles may reduce pore connectivity under loading.

The higher velocities observed in M1_R compared to M3_R are attributed to its dual composition: M1_R contains both rubber powder (RTWP) and rubber granulate (RTWG), while M3_R contains only rubber powder. The presence of larger granulate particles in M1_R enhances pore interconnectivity and promotes higher flow rates. This highlights the importance of both the proportion and the size distribution of rubber particles in controlling permeability [55].

Another thing to bear in mind is the flow regime. The flow regime can transition between laminar and turbulent as the velocity increases and the pore structure becomes more complex [56]. This can influence the overall flow behavior in the case of fRCA–RTW blends.

In this study, the M1_R test results reveal the existence of non-Darcian flow behavior. As shown in Figure 9 (inset), seepage does not commence proportionally to the hydraulic gradient but begins only after a certain threshold gradient (i_t_) is exceeded. Below this threshold, the flow velocity decreases dramatically, indicating a pre-linear filtration regime. This behavior is consistent with the concept of a threshold gradient (i_0_) frequently reported in soils with irregular pore structures [57].

In contrast, the permeable behavior of the M3 and M3_R mixes follows Darcy’s law more closely (Figure 9, main plot). For these materials, the velocity of fluid flow is approximately proportional to the hydraulic gradient across the tested range, as expected in Darcian flow (see the details of the proposed linear function summarized in Table 5).

For the mixture containing 20% rubber waste (M1_R), however, two distinct phases of filtration can be identified:

1. Pre-linear filtration, where the relationship between velocity and gradient is curved, reflecting a gradual increase in permeability as interconnected pore channels open under increasing hydraulic forces.
2. Linear filtration, where the flow aligns more closely with Darcy’s law at higher gradients.

This phenomenon arises from the complex pore structure introduced by the combination of rubber powder and granulate in M1_R, which alters pore connectivity and requires a minimum driving force for steady flow to establish. Sas et al. [58] observed similar trends in recycled aggregates, although under lower consolidation pressures (10 kPa) and smaller gradients.

These findings highlight the need for further investigations at smaller hydraulic gradients (*i* < 1) to verify or reject the applicability of Darcy’s law to recycled concrete aggregate–rubber mixtures. Such tests would help to more precisely identify threshold gradients and clarify the transition between non-Darcian and Darcian regimes.

While the observed deviations from linear Darcy behavior—particularly in the M1_R blend—suggest the presence of non-Darcian flow regimes, especially at higher hydraulic gradients, the current study did not extend to modeling this behavior using established non-linear flow models such as the Forchheimer equation. The Forchheimer model, which incorporates an inertial (quadratic) term to account for deviations at high velocities or in highly porous media, has been successfully applied in previous research on recycled aggregates and rubber-modified systems with complex pore geometries [24,58]. However, in this investigation, the primary focus was on assessing the influence of rubber content and compaction pressure on permeability behavior, rather than developing a comprehensive rheological model of the flow regime.

Furthermore, limitations related to the experimental design—including the range of hydraulic gradients applied and the accuracy of velocity measurements at low gradients—constrained the reliable identification of the critical transition point between Darcian and non-Darcian flow. Future studies are recommended to include extended gradient ranges (*i* < 1 and *i* > 20), refined measurement protocols, and application of empirical models such as the Forchheimer or Hazen–Dupuit–Darcy equations to more accurately characterize non-linear flow in fRCA–RTW systems.

### 3.2. RTWS

**Figure 10:**
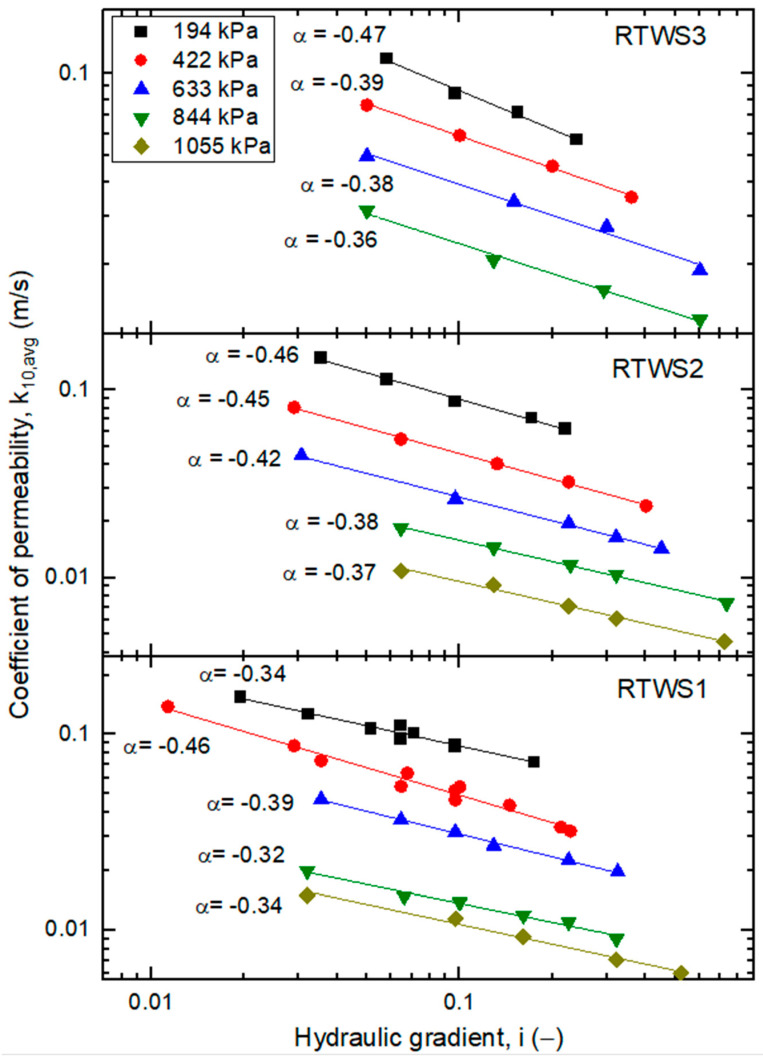
Variation of *k*_10,*avg*_ with *i* corresponding to varied *p*’ for tire shreds (RTWS mixtures) (*p*’-values are given in the legend).

In Figure 10, the average permeability coefficient (*k*_10,avg_) of compressed shredded tire specimens (RTWS1–3) is plotted against the hydraulic gradient. The results show considerably higher permeability values compared to the RCA-based mixtures. Specifically, *k*_10,*avg*_ ranges from approximately 6.0 × 10^−3^–1.5 × 10^−1^ m/s for RTWS1, 4.6 × 10^−3^–1.5 × 10^−1^ m/s for RTWS2, and 1.3 × 10^−2^–1.1 × 10^−1^ m/s for RTWS3. These values are consistent with those typically found in well-drained granular soils, such as gravels [50].

The higher permeability of RTWS compared to RCA–RTW mixtures is a consequence of both the larger specimen size tested and the open pore network generated by shredded tire particles. Furthermore, the three RTWS specimens differed not only in particle dimensions but also in the construction of the specimens. RTWS1 was composed of the smallest particles (mean ≈ 3.0 mm), RTWS2 of slightly larger particles (≈3.65 mm), and RTWS3 of the coarsest particles (up to ≈5.3 mm) (see Table 4). As expected, larger rubber pieces promoted faster flow, explaining why RTWS3 showed the highest flow velocities and less sensitivity to applied pressure.

A clear dependence of *k*_10,*avg*_ on the hydraulic gradient is visible. For all RTWS mixtures, permeability decreases as the hydraulic gradient increases, following a power–law relationship of the form:

(7) $$k_{10,avg}\propto a\cdot i^{\alpha },$$

where the slope coefficients (α) are between −0.32 and −0.47, depending on the sample and applied pressure (Figure 10). The reduction in permeability is significant, ranging from approximately 56% to 63%. This effect can be attributed to changes in the flow regime: at low gradients, flow is dominated by laminar seepage, whereas at higher gradients, turbulence and flow inhibition occur in the irregular voids between tire shreds. This observation is comparable to the findings reported for the M3 mixture, pure fine recycled concrete aggregate. Mixtures composed of only one material, such as aggregate or rubber, behave similarly. Mixtures composed of two components do not demonstrate analogous behavior.

The results also suggest the existence of a threshold gradient. For RTWS, once the hydraulic gradient exceeds approximately *i* = 0.2–0.3, further increases in *i* have little effect on *k*_10,*avg*_. Beyond this range, permeability values stabilize across all confining pressures. This threshold behavior is consistent with earlier observations for pure RCA mixtures (e.g., M3) and has also been reported by Głuchowski et al. [24].

The applied compression pressure also strongly influences permeability. At low hydraulic gradients, the difference in *k*_10,*avg*_ between the lowest and highest pressures reaches 60% for RTWS1 and RTWS2 but decreases to only ~20–25% at high pressures (>800 kPa). For RTWS3, the effect of pressure is minimal, and permeability values remain nearly constant across the range of applied stresses, reflecting the dominance of large voids created by the coarser rubber pieces.

Finally, the results indicate that beyond approximately 800 kPa, no further reduction in permeability occurs for any of the RTWS mixtures. In all cases, the decrease in *k*_10,*avg*_ with increasing gradient and confining pressure is well described by power functions, with coefficients of determination (R^2^) close to unity.

**Figure 11:**
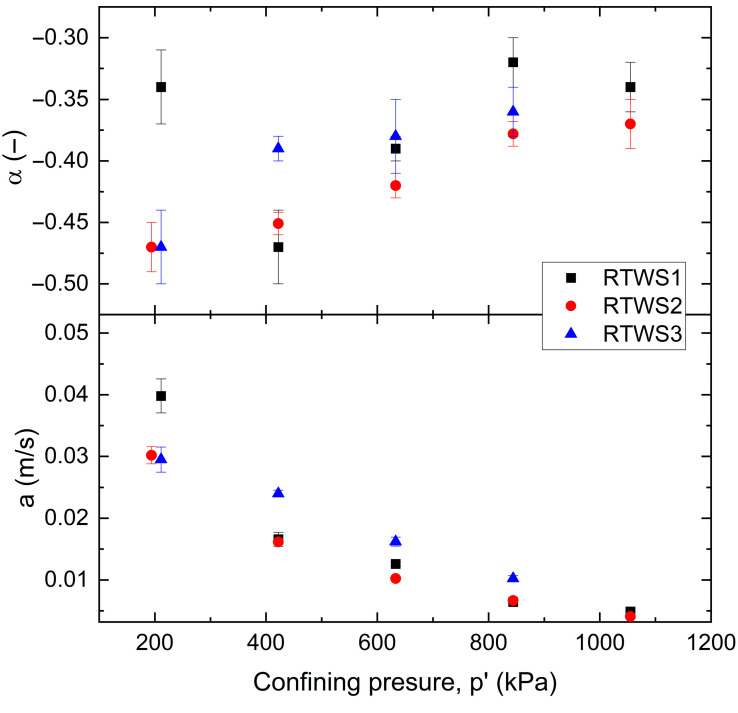
Variation in coefficient *a* and *α* with *p*’ for the tested RTWS blend.

In Figure 11, the dependence of the power function coefficients (α—top plate, a—bottom plate) on confining pressure is shown. For all RTWS mixtures, *α* tends to become less negative (i.e., increases) as confining pressure increases. The RTWS1 mix (black squares) shows the highest values of *α* across most of the range. The RTWS2 (red circles) and RTWS3 (blue triangles) blends show similar trends but start at more negative *α* values. Error bars indicate uncertainty or variability in the measurements. The parameter *a* decreases with increasing confining pressure for all three RTWS samples. The RTWS1 mix consistently shows slightly higher values of *a* at lower confining pressures. The RTWS2 and RTWS3 blends, on the contrary, follow similar trends, with RTWS2 generally showing the lowest values at higher pressures.

## 4. Conclusions

To gain a more comprehensive understanding of recycled aggregates, mainly concrete and rubber aggregates, and further promote their application in practical engineering, experimental investigations into the water permeability property of fRCA, fRCA–RTW mixtures, and RTWS blends are carried out in this study. The main conclusions are as follows:

- The tested crushed concrete aggregate has a filtration coefficient corresponding to semi-permeable, cohesive materials, e.g., clay. The filtration coefficient was found to be in the range of 10^−6^ to 10^−7^ m/s. This is undoubtedly associated with the composition of fine fractions, with a proportion of 20% in the M3 mixture.
- There is a non-linear relationship between the average coefficient of permeability of fRCA and hydraulic gradient: the former decreases as the latter increases. This may indicate the occurrence of clogging and obstruction of water flow caused by high flow velocities.
- No effect of consolidation pressure on fRCA filtration parameters was noted, although the tests were performed at low pressures. The experiments could be extended to higher pressures, as in the case of pure rubber waste.
- For fRCA, a linear increase in flow velocity was observed as the gradient increased. No characteristic filtration phases were observed, i.e., a pre-linear phase, a transition phase, and a linearisation phase. The initial gradient could not be determined, which may be related to the excessive gradients used in the studies.
- The addition of tire rubber waste modifies the permeability conditions of fRCA. In the mixtures, M1_R and M3_R, rubber was used as a partial replacement for coarser concrete aggregates, resulting in increased porosity and permeability. It is an increase of one order of magnitude. The presence of fine fractions in crushed concrete in the mixtures remains a concern.
- The higher the rubber content in the mixture (e.g., 20%), the more visible the division into two regimes: a low-pressure, aggregate-dominated regime where pore closure reduces flow, and a high-pressure, rubber-dominated regime where macropores associated with rubber particles increase flow; this two-phase behavior is absent in mixtures without rubber.
- The mixtures containing rubber (M1_R and M3_R) demonstrate significantly higher permeability coefficients at lower effective stresses compared to pure fRCA. The effect of consolidation for these materials is visible. There is a migration of finer particles and rubber particles into the pore spaces. The obtained hydraulic conductivity is a function of flow pressure and effective stress, especially for the M1_R mix.
- For the fRCA–RTW mixtures, an increase in flow velocity can be observed with an increase in the hydraulic gradient. The M1_R blend with 20% of rubber content exhibits the non-Darcian flow of water with threshold gradient occurrence. The M3_R mix, with a lower rubber content, i.e., 10%, performs more like pure fRCA and behaves according to Darcy’s law.
- Pure shredded tires in all studied cases, even at the highest possible compression pressure of 1055 kPa, are characterized by a high filtration coefficient similar to the water permeability of coarse-grained soils. This coefficient depends exponentially on the hydraulic gradient, i.e., the greater the gradient, the lower the filtration capacity. This is due to the uneven increase in water speed through the voids of the tire pieces, caused by a change in flow regime from laminar to turbulent.
- The influence of the water pressure height gradient depends on the compression pressure of the RTWS specimens. At a pressure greater than 800 kPa, the gradient *i* has little effect on the permeability coefficient.

However, several limitations were identified that can inform and guide future research:

- Restricted hydraulic gradient range. Future studies should consider applying a broader range of gradients (e.g., *i* < 1 to *i* > 20) to better define the transition between laminar and turbulent flow regimes.
- Absence of non-linear flow modeling. Future work should integrate such models like the Forchheimer or Hazen–Dupuit–Darcy to improve the interpretation and predictive capacity of filtration parameters.
- Limited consolidation pressures. Investigation at higher pressures—particularly for mixed and granular systems—would enhance the practical relevance of the findings.
- Lack of microstructural characterization. This study did not include microstructural analyses such as Scanning Electron Microscopy (SEM), Mercury Intrusion Porosimetry (MIP), or X-ray Computed Tomography (CT). Such techniques could elucidate key mechanisms influencing permeability, including pore connectivity, rubber–cement interaction, and clogging phenomena. Incorporating microstructural tools in future research would enable a more comprehensive understanding of how internal structure affects hydraulic behavior.
- Material heterogeneity and rubber content variability. Future experiments could benefit from controlled gradation of rubber particles and standardized RTW material specifications to reduce experimental scatter and isolate the effects of particle shape, size, and composition.
- Absence of temperature and/or saturation effect. Investigations could be extended to study thermal effects and unsaturated flow conditions, which would better reflect more realistic service environments, particularly in field conditions characterized by fluctuating temperatures and partial saturation.

To sum up, the experimental findings provide a comprehensive overview of the hydraulic behavior of recycled materials, highlighting their limitations and potential. The crushed concrete aggregate (fRCA) exhibits low permeability, suggesting a dense microstructure. The introduction of rubber tire waste (RTW) increases porosity and facilitates water transmission, especially in the M1_R and M3_R blends. RTW acts as an effective pore-forming agent, although with growing heterogeneity. In the case of pure rubber specimens (RTWS), the permeability reflects a granular framework, suitable for drainage or lightweight fill applications. However, permeability decreases under higher gradients, suggesting instability and turbulence. While the permeability of these recycled materials varies, the results confirm their feasibility in geotechnical and environmental applications. RTW can be tailored through material selection and compaction, making these composites promising candidates for sustainable infrastructure solutions.

## Figures and Tables

**Table 1:** Previous studies on RCA and RTW permeability.

Sample/Material	Research Content	Major Conclusion	Reference
RCA	Pore characterization of manufactured aggregates	High roughness grains with irregular shapes complicate the flow path of water.	[18]
RCA	Porosity of recycled concrete with the substitution of RCA	Void ratio, porosity, and, therefore, hydraulic conductivity of RCA are higher than natural aggregates.	[19]
RCA	Water absorption of recycled aggregates—new approach	Patches of cement paste attached to the surface of recycled aggregate may affect water absorption in a manner different from conventional aggregate. Because of this, the standard duration of 24 h of saturation is not suitable for RCA.	[20]
RCA	Geotechnical and geoenvironmental properties of recycled construction and demolition materials	The coefficient of permeability is equal to 3.3 × 10^−8^ m/s, which is similar to cohesive soils and materials used for dam and levee cores.	[21]
RCA	Replacement of RCA by crushed clay brick as an unbound road sub-base	The hydraulic conductivity of RCA ranges from 2.04 × 10^−3^ m/s to 2.67 × 10^−3^ m/s.	[22,23]
RCA	Permeability and leaching properties of RCA	The average coefficient of permeability for linear flow in RCA is equal from ~1.02 × 10^−4^ m/s to ~2.08 × 10^−5^ m/s for aggregates from 0.05–16 mm to 0–8 mm, respectively. RCA proves its good quality as a permeable material.	[24]
Tire shreds	Shredded tires as lightweight fill backfill material for retaining structures	Tire shreds have a hydraulic conductivity of ~3 × 10^−2^ cm/s, indicating that they are well suited for use as drainage material in landfill leachate collection systems.	[25]
Tire shreds	Evaluation of the permeability of tire shreds under vertical loading	Despite experiencing large axial strains, the average permeability of the tire shred sample consistently remained two to three orders of magnitude higher than the design performance criterion of 0.01 cm/s for landfill drainage layers, suggesting that the compressible nature of tire shreds will not interfere with their use as a leachate collection drainage layer in municipal solid waste landfills.	[26]
Crumb rubber particles and ballast aggregates	The effect of size and percentage of crumb rubber particles on hydraulic conductivity of rubber-modified railway ballast	The nonlinear relationship between applied hydraulic gradient and water flow velocity is observed for different characterized conditions. Rubber-modified ballast under extreme conditions (using a higher percentage of smaller-sized CR particles) shows a flow regime between laminar and turbulent (with n value around 0.8).	[27]
Rubberized pervious concretes	Permeability of rubberized plain pervious concrete	Permeability is significantly reduced with tire rubber. A 10% rubber replacement diminished the permeability coefficient by 28%.	[28]
Pervious concrete (binary blended)	Characteristics of pore structure and permeability prediction, with partial fly ash replacement (10–20%)	Image-based porosity (area fraction) slightly exceeds volumetric porosity. Pore characteristics (via morphological techniques) agree across methods, with a strong match between experimental and predicted permeability.	[29]
Pervious concrete (aggregate mix)	Evaluation of pore size distribution and permeability reduction behaviour	Lower aggregate-to-cement ratio and higher water-to-cement ratio decrease porosity and permeability but increase compressive strength, thermal conductivity, and abrasion resistance; porosity, permeability, and compressive strength correlate via a power–law.	[30,31]
Rubberized concrete (RC)	Prediction of compressive strength using an evolved random forest model (RF optimized by BAS algorithm)	High predictive accuracy (correlation coefficient ≈ 0.96). Key influencing variables: RC age (most significant), followed by water–cement ratio, fine rubber aggregate content, coarse rubber aggregate content, and coarse aggregate.	[32]
Jet-grouting coalcretes (coalcrete)	Optimized neural network (BPNN tuned via BAS) to predict unconfined compressive strength (UCS)	The BAS-optimized BPNN model outperforms MLR, LR, and SVM in accuracy and reliability. Sensitivity analysis shows curing time is the most critical variable, followed by water–cement ratio and coal–grout ratio.	[33]

**Table 2:** Plan of experimental work.

Mixture fRCA–RTW	Cell Pressure	Effective Stress	Hydraulic Gradient
	kPa	kPa	-
M3	210	10	1, 2, 4, 8, 12, 16
	220	20	1, 2, 4, 8, 12, 16
	240	40	1, 2, 4, 8, 12, 16
	280	80	1, 2, 4, 8, 12, 16
	320	120	1, 2, 4, 8, 12, 16
M1_R; M3_R	220	20	2, 4, 6, 8, 10, 12, 16
	240	40	2, 4, 6, 8, 10, 12, 16
	260	60	2, 4, 6, 8, 10, 12, 16
	280	80	2, 4, 6, 8, 10, 12, 16
	320	120	2, 4, 6, 8, 10, 12, 16

**Table 3:** Input flow pressures list.

Mixture fRCA–RTW	Top Pressure	Base Pressure	Input Pressure	Hydraulic Gradient
	kPa	kPa	kPa	-
M3-1	199.2	199.7	0.5	1
M3-2	199.1	200.6	1.5	2
M3-3	200.0	203.2	3.2	4
M3-4	200.3	206.5	6.2	8
M3-5	200.0	209.0	9.0	12
M3-6	200.0	212.0	12.0	16
M1_R-1	200.0	201.4	1.4	2
M1_R-2	200.0	202.8	2.8	4
M1_R-3	200.0	204.2	4.2	6
M1_R-4	200.0	205.6	5.6	8
M1_R-5	200.0	207.0	7.0	10
M1_R-6	200.0	208.4	8.4	12
M1_R-7	200.0	211.2	11.2	16
M3_R-1	200.0	201.4	1.4	2
M3_R-2	200.0	202.8	2.8	4
M3_R-3	200.0	204.2	4.2	6
M3_R-4	200.0	205.6	5.6	8
M3_R-5	200.0	207.0	7.0	10
M3_R-6	200.0	208.4	8.4	12
M3_R-7	200.0	211.2	11.2	16

**Table 4:** Details of the tire shreds samples and testing procedure.

Tire Shreds Sample	Length	Width	Thickness	Total Sample Mass	Compression Stress	Hydraulic Gradient
	mm	mm	mm	kg	kPa	-
RTWS1	260	140	16	12.3	211/422/633/844/1055	~0.02–0.52
RTWS2	250	90	17	13.1	194/422/633/844/1055	~0.04–0.72
RTWS3	470	145	16	11.0	211/422/633/844	~0.05–0.60

**Table 5:** Details of the linear filtration phase for all tested fRCA–RTW mixtures.

Mixture fRCA–RTW	M1_R	M3_R	M3
y = ax + b			
a (10^−6^ m/s)	1.6 ± 0.3	6.2 ± 0.5	1.15 ± 0.03
b (10^−6^ m/s)	3.0 ± 2.0	2.1 ± 0.4	0.20 ± 0.003

## Data Availability

The experimental data that support the findings of this study are available in RepOD with the identifier: DOI: 10.18150/OB8OVR.

## References

[1] Abbass K., Qasim M.Z., Song H., Murshed M., Mahmood H., Younis I. (2022). A review of the global climate change impacts, adaptation, and sustainable mitigation measures. Environ. Sci. Pollut. Res..

[2] Schilling M., Chiang L. (2011). The effect of natural resources on a sustainable development policy: The approach of non-sustainable externalities. Energy Policy.

[3] A/RES/70/1-Transforming Our World: The 2030 Agenda for Sustainable Development. https://sdgs.un.org/2030agenda.

[4] Silva Vieira C., Pereira P., De Lurdes Lopes M. (2016). Recycled Construction and Demolition Wastes as filling material for geosynthetic reinforced structures. Interface properties. J. Clean Prod..

[5] Asanousi Lamma O. (2021). The impact of recycling in preserving the environment. Int. J. Appl. Res..

[6] Kumbhar S., Gupta A., Desai D. (2013). Recycling and Reuse of Construction and Demolition Waste for Sustainable Development. OIDA Int. J. Sustain. Dev..

[7] Moussa A., El Naggar H., Sadrekarimi A. (2023). Dynamic characterization of tire derived aggregates using cyclic simple shear and bender element tests. Soil Dyn. Earth Eng..

[8] Farooq M.A., Nimbalkar S., Fatahi B. (2022). Sustainable Applications of Tyre-Derived Aggregates for Railway Transportation Infrastructure. Sustainability.

[9] Kumar P., Mina U. (2021). Fundamentals of Ecology and Environment.

[10] Dzięcioł J., Radziemska M. (2022). Blast Furnace Slag, Post-Industrial Waste or Valuable Building Materials with Remediation Potential?. Minerals.

[11] Khan A.A., Balunaini U., Costa A., Nguyen N.H.T. (2024). A review on sustainable use of recycled construction and demolition waste aggregates in pavement base and subbase layers. Clean. Mater..

[12] Punetha P., Nimbalkar S. (2025). Utilisation of construction and demolition waste and recycled glass for sustainable flexible pavements: A critical review. Transp. Geotech..

[13] Chen S.H. (2015). Hydraulic Structures.

[14] Elhakim A.F. (2016). Estimation of soil permeability. Alex. Eng. J..

[15] Malinowska E., Szymański A., Sas W. (2010). Estimation of Flow Characteristics in Peat. Geotech. Test. J..

[16] Sobolewski M. (2011). Various methods of the measurement of the permeability coefficient in soils-possibilities and application. EJPAU.

[17] (2024). Standard Test Method for Measurement of Hydraulic Conductivity of Saturated Porous Materials Using a Flexible Wall Permeameter.

[18] Deshpande Y.S., Hiller J.E. (2011). Pore characterization of manufactured aggregates: Recycled concrete aggregates and lightweight aggregates. Mater. Struct..

[19] Gómez-Soberón J.M. (2002). Porosity of recycled concrete with substitution of recycled concrete aggregate: An experimental study. Cem. Concr. Res..

[20] Tam V.W.Y., Gao X.F., Tam C.M., Chan C.H. (2008). New Approach in Measuring Water Absorption of Recycled Aggregates. Constr. Build. Mater..

[21] Arulrajah A., Piratheepan J., Disfani M.M., Bo M.W. (2013). Geotechnical and geoenvironmental properties of recycled construction and demolition materials in pavement subbase applications. J. Mater. Civ. Eng..

[22] Poon C.S., Qiao X.C., Chan D. (2006). The cause and influence of self-cementing properties of fine recycled concrete aggregates on the properties of unbound sub-base. Waste Manag..

[23] Poon C.S., Chan D. (2006). Feasible use of recycled concrete aggregates and crushed clay brick as unbound road sub-base. Constr. Build. Mater..

[24] Głuchowski A., Sas W., Dzięcioł J., Soból E., Szymański A. (2019). Permeability and Leaching Properties of Recycled Concrete Aggregate as an Emerging Material in Civil Engineering. Appl. Sci..

[25] Gonzales L., Williams J. (1996). Use of Shredded Tires as Lightweight Fill Backfill Material for Retaining Structures. Waste Manag. Res..

[26] Warith M.A., Evgin E., Benson P.A.S., Rao S.M. (2005). Evaluation of Permeability of Tire Shreds Under Vertical Loading. J. Test. Eval..

[27] Koohmishi M., Azarhoosh A. (2020). Hydraulic conductivity of fresh railway ballast mixed with crumb rubber considering size and percentage of crumb rubber as well as aggregate gradation. Const. Build. Mater..

[28] Gesoğlu M., Güneyisi E., Khoshnaw G., İpek S. (2014). Investigating properties of pervious concretes containing waste tire rubbers. Constr. Build. Mater..

[29] Maguesvari Muthaiyan U. (2024). Characteristics of pore structure and permeability prediction in binary blended pervious concrete. Matéria (Rio J.).

[30] Huang J., Zhang Y., Sun Y., Ren J., Zhao Z., Zhang J. (2021). Evaluation of pore size distribution and permeability reduction behavior in pervious concrete. Const. Build. Mater..

[31] Lyu Q., Dai P., Chen A. (2024). Correlations among physical properties of pervious concrete with different aggregate sizes and mix proportions. Road Mater. Pavement Des..

[32] Sun Y., Li G., Zhang J., Qian D. (2019). Prediction of the Strength of Rubberized Concrete by an Evolved Random Forest Model. Adv. Civ. Eng..

[33] Sun Y., Zhang J., Li G., Wang Y., Sun J., Jiang C. (2019). Optimized neural network using beetle antennae search for predicting the unconfined compressive strength of jet grouting coalcretes. Int. J. Numer. Anal. Methods Geomech..

[34] Mitchell J.K., Soga K. (1993). Fundamentals of Soil Behavior.

[35] Nedeljković M., Visser J., Šavija B., Valcke S., Schlangen E. (2021). Use of fine recycled concrete aggregates in concrete: A critical review. J. Build. Eng..

[36] Villagrán-Zaccardi Y., Broodcoorens L., Van den Heede P., De Belie N. (2023). Fine Recycled Concrete Aggregates Treated by Means of Wastewater and Carbonation Pretreatment. Sustainability.

[37] Gabryś K., Radzevičius A., Szymański A., Šadzevičius R. (2021). Shear Strength Characteristics of Recycled Concrete Aggregate and Recycled Tire Waste Mixtures from Monotonic Triaxial Tests. Materials.

[38] Gabryś K., Šadzevičius R., Dapkienė M., Ramukevičius D., Sas W. (2023). Effect of a Fine Fraction on Dynamic Properties of Recycled Concrete Aggregate as a Special Anthropogenic Soil. Materials.

[39] Gabryś K. (2023). Geomechanical Characterization of Crushed Concrete–Rubber Waste Mixtures. Sustainability.

[40] (2017). Rozpoznanie i Badania Geotechniczne–Oznaczanie i Klasyfikowanie Gruntów–Część 2: Zasady Klasyfikowania (Geotechnical Investigation and Testing–Determination and Classification of Soils–Part 2: Classification Principles).

[41] Humboldt Mfg. Co (2011). Humboldt HM-4150-40-60 Manual: Vol. Testing Equipment for Construction Materials.

[42] Raghunandan M.E., Juneja A., Benson Hsiung B.C. (2016). Preparation of reconstituted sand samples in the laboratory. Int. J. Geotech. Eng..

[43] Kandalai S., Singh P.N., Singh K.K. (2018). Permeability of granular soil employing flexible wall permeameter. Arab. J. Geosci..

[44] Fell R., MacGregor P., Stapledon D., Bell G. (2005). Geotechnical Engineering of Dams.

[45] Azram A.M., Cameron D.A. (2013). Geotechnical properties of blends of Recycled Clay Marsony and Recycled Concrete Aggregates in Unbound Pavement Construction. J. Mater. Civ. Eng..

[46] Arulrajah A., Piratheepan J., Ali M.M.Y., Bo M.W. (2012). Geotechnical properties of recycled concrete aggregate in pavement sub-base applications. Geotech. Test. J..

[47] McCulloch T., Kang D., Shamet R., Lee S.J., Nam B.H. (2017). Long-Term performance of recycled concrete aggregate for subsurface drainage. J. Perform. Constr. Facil..

[48] Zhu Z., Zhou M., Wang B., Xu X. (2024). Enhancing permeability and mechanical properties of rubber cement-based materials through surface modification of waste tire rubber powder. Const. Build. Mater..

[49] Liu H., Luo G., Gong Y., Wei H. (2018). Mechanical Properties, Permeability, and Freeze–Thaw Resistance of Pervious Concrete Modified by Waste Crumb Rubbers. Appl. Sci..

[50] Das B.M. (1990). Principles of Geotechnical Engineering.

[51] Chapuis R.P. (1992). Similarity of internal stability criteria for granular soils. Can. Geotech. J..

[52] Chapuis R.P. (2012). Predicting the saturated hydraulic conductivity of soils: A review. Bull. Eng. Geol. Environ..

[53] Daniel D.E., Daniel D.E., Trautwein S.J. (1994). State of the art: Laboratory hydraulic conductivity tests for saturated soils. Hydraulic Conductivity and Waste Contaminant Transport in Soil.

[54] Wang F., Sun C., Zhao H., Liu Y. (2023). Effect of waste tire rubber crumb on impermeability of cement-stabilized soil. Environ. Technol..

[55] Akhtar A.Y., Tsang H.H. (2024). Dynamic leaching assessment of recycled polyurethane-coated tire rubber for sustainable engineering applications. Chem. Eng. J..

[56] Fiorillo F., Esposito L., Ginolfi M., Leone G. (2024). New Insights into Turbulent and Laminar Flow Relationships Using Darcy–Weisbach and Poiseuille Laws. Water.

[57] Hansbo S. (2001). Consolidation equation valid for both Darcian and non-Darcian flow. Géotechnique.

[58] Sas W., Dzięcioł J., Głuchowski A. (2019). Estimation of Recycled Concrete Aggregate’s Water Permeability Coefficient as Earth Construction Material with the Application of an Analytical Method. Materials.

